# Assessment and optimization of respiratory syncytial virus prophylaxis in Connecticut, 1996–2013

**DOI:** 10.1038/s41598-021-90107-8

**Published:** 2021-05-21

**Authors:** Ben Artin, Virginia E. Pitzer, Daniel M. Weinberger

**Affiliations:** Department of Epidemiology of Microbial Diseases, Yale School of Public Health, Yale University, New Haven, USA

**Keywords:** Viral infection, Epidemiology, Paediatric research

## Abstract

Respiratory syncytial virus (RSV) causes seasonal respiratory infection, with hospitalization rates of up to 50% in high-risk infants. Palivizumab provides safe and effective, yet costly, immunoprophylaxis. The American Academy of Pediatrics (AAP) recommends palivizumab only for high-risk infants and only during the RSV season. Outside of Florida, the current guidelines do not recommend regional adjustments to the timing of the immunoprophylaxis regimen. Our hypothesis is that adjusting the RSV prophylaxis regimen in Connecticut based on spatial variation in the timing of RSV incidence can reduce the disease burden compared to the current AAP-recommended prophylaxis regimen. We obtained weekly RSV-associated hospital admissions by ZIP-code in Connecticut between July 1996 and June 2013. We estimated the fraction of all Connecticut RSV cases occurring during the period of protection offered by immunoprophylaxis (“preventable fraction”) under the AAP guidelines. We then used the same model to estimate protection conferred by immunoprophylaxis regimens with alternate start dates, but unchanged duration. The fraction of RSV hospitalizations preventable by the AAP guidelines varies by county because of variations in epidemic timing. Prophylaxis regimens adjusted for state- or county-level variation in the timing of RSV seasons are superior to the AAP-recommended regimen. The best alternative strategy yielded a preventable fraction of 95.1% (95% CI 94.7–95.4%), compared to 94.1% (95% CI 93.7–94.5%) for the AAP recommendation. In Connecticut, county-level recommendations would provide only a minimal additional benefit while adding complexity. Initiating RSV prophylaxis based on state-level data may improve protection compared with the AAP recommendations.

## Introduction

RSV causes upper respiratory tract infections in people of all ages. In adults, RSV infection is typically asymptomatic or gives rise to mild respiratory illness. However, RSV infection often incites more serious respiratory illness in children, with hospitalization rates as high as 4.4% in infants with no comorbidities, making RSV a leading cause of hospitalization among infants^1–4^. There is also an underdiagnosed burden of disease in the elderly, and adults can act as a reservoir of RSV transmission in the population^5^.

For infants, risk factors of serious illness and hospitalization due to RSV infection include prematurity, chronic lung disease of prematurity, congenital heart disease, anatomic pulmonary abnormalities, neuromuscular disorders, trisomy 21, and immunocompromised status^6^. The hospitalization rate due to RSV among high-risk infants is anywhere from two to ten times higher than among infants with no comorbidities^4^.

A monoclonal antibody (palivizumab) is available in the US for prevention of RSV infection in high-risk infants less than 2 years old. Palivizumab is administered via intramuscular injection and requires five monthly doses, priced at $1500–$3000 per dose^7,8^. Palivizumab has been found to be effective in reducing hospitalizations due to RSV; in double-blinded trials in high-risk infants, it reduced the hospitalization rate by 45–55% compared to placebo^9,10^. It has also been found to be safe and well-tolerated^9^. Owing to the high cost, the 2014 revision of the AAP RSV prophylaxis guidelines recommends palivizumab only for high-risk infants and only during the RSV season^6,11,12^.

RSV seasonality and spatial variability have been established throughout the United States, based on the CDC’s National Respiratory and Enteric Virus Surveillance System data; RSV has been found to have an annual or biennial cycle with regionally variable seasonal onset ranging from late August to mid-December and offset ranging from mid-March to late April^13–15^. In Connecticut, previous analysis of RSV-associated hospital admissions has shown spatial variability in the timing of peak RSV incidence, with epidemics occurring earlier in large urban areas and later in sparsely-populated rural areas^16^. The AAP acknowledges the potential significance of spatial and temporal variation in RSV incidence, but—outside of Florida, whose RSV season leads most of the rest of the nation by approximately 2 months—does not recommend local variance from nationwide prophylaxis guidelines^6^.

Motivated by the AAP’s recognition of the significance of spatial variation in formulating prophylaxis guidelines and the known spatial variability of RSV incidence in Connecticut, we aim to determine whether protection due to RSV prophylaxis would be enhanced by having localized guidelines for the initiation of prophylaxis by addressing the following questions:

- To what extent does county-level variability of RSV incidence in Connecticut impact the effectiveness of the AAP-recommended prophylaxis regimen?
- Would county- or state-specific alternative regimens increase the benefits of prophylaxis in comparison to the AAP-recommended regimen without increasing the total cost of the regimen?
- Is there important year-to-year variation in timing of RSV season in Connecticut?

## Methods

### Source of data

We obtained data consisting of all hospitalizations among children < 2 years old in Connecticut from 1996 to 2013 from the Connecticut State Inpatient Database through the Connecticut Department of Public Health (CT DPH). The relevant variables in the data were ZIP code of residence, patient age, ICD-9 defined diagnoses (up to 10 diagnoses per hospital admission), and week and year of hospital admission. The study was approved by the Human Investigation Committees at Yale University and the CT DPH. The authors assume full responsibility for analyses and interpretation of the data obtained from CT DPH.

### Definitions

We defined an RSV-associated hospitalization in Connecticut to be a hospital stay for which:

- the postal code of residence listed the in patient’s medical record was in Connecticut, and
- the hospital stay was associated with ICD-9-CM diagnosis code 079.6 (RSV), 466.11 (acute bronchiolitis due to RSV), or 480.1 (pneumonia due to RSV).

We designated epidemiological years as beginning in the first full week of July, in order to accommodate the timing of RSV peaks (which span two calendar years). Due to changes in diagnostic coding, we excluded data prior to July 1996; we similarly excluded data after June 2013 due to incompleteness of data for the 2013–14 epidemiological year. Using the weekly hospitalization data, we identified 9701 RSV-associated hospitalizations among 300,693 total hospitalizations of patients < 2 years old in Connecticut between July 1996 and June 2013 (inclusive).

### Spatial aggregation of RSV incidence

**Figure 1:**
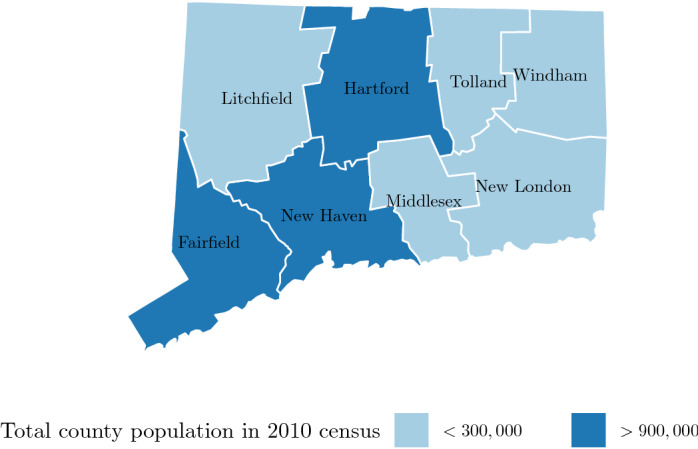
Subdivision of Connecticut into high-population and low-population counties *Figure created using* ggplot2 * version 3.3.2* (https://ggplot2.tidyverse.org).

We analyzed RSV data aggregated either to the state level or into four geographic areas:

- One area for each of the counties with high population levels and high RSV case counts: Hartford, Fairfield, and New Haven (Fig. 1).
- One discontiguous area comprised of all remaining counties in Connecticut: Tolland, Windham, Middlesex, Litchfield, and New London (“low-population counties”).

We chose county-level aggregation with an eye towards using this data to inform clinical practice guidelines, which are typically based on administrative areas for reasons of administrative and implementation simplicity. We chose to aggregate low-population counties into a single area because our analysis in those counties individually yielded results with low statistical power.

### Analysis approach

Our goal was to construct a model of annual RSV incidence that allowed us to estimate characteristics of RSV seasons, such as season onset, season offset, and preventable fraction of a prophylaxis regimen. To that end, we fit smooth curves through annual RSV hospitalization data and used resampling to obtain confidence intervals. This was performed using the pspline.inference package in R, which we developed for this purpose^17,18^.

We defined season onset as the time when cumulative incidence rises beyond 2.5% of the total cumulative incidence, and season offset as the time when cumulative incidence rises beyond 97.5% of the total cumulative incidence for an epidemiological year. We aggregated our data by surveillance week across all surveillance years, and estimated RSV season onset and offset for each county.

The choice of 2.5% and 97.5% incidence thresholds was based on our intent to use estimates of RSV season onset and offset to evaluate alternative prophylaxis regimens (as described below), while maintaining the 5-month dosing schedule (and therefore not increasing medication cost). Incidence cutoffs other than 2.5% and 97.5% were briefly investigated, but quickly ruled out because they produced prophylaxis regimens starting long prior to RSV season onset or ending long after RSV season offset, and therefore had very low preventable fractions.

### Evaluating alternative prophylaxis regimens

We compared the current AAP guidelines with six alternative prophylaxis regimens:

- *By current AAP national guidelines:* These guidelines recommend RSV prophylaxis with 5 monthly doses of palivizumab, starting in November, for all high-risk infants in their first year of life; in the second year of life, palivizumab is only recommended for some subgroups. For simplicity, we assumed that protection by palivizumab begins on November 15th.
- *By statewide onset:* Prophylaxis administration begins at statewide median RSV season onset.
- *By statewide midpoint:* Prophylaxis administration begins 12 weeks before the average of the statewide median season onset and offset.
- *By statewide offset:* Prophylaxis administration begins 24 weeks before the statewide median RSV season offset.
- *By county-level onset:* Prophylaxis administration begins at county-level median RSV season onset.
- *By county-level midpoint:* Prophylaxis administration begins 12 weeks before the average of the county-level median RSV season onset and offset.
- *By county-level offset:* Prophylaxis administration begins 24 weeks before the county-level median RSV season offset.

In all these regimens, prophylaxis consists of five monthly doses of palivizumab, and protection is assumed to last 24 weeks from the first dose. We rounded each alternative regimen to the nearest week to more closely reflect how prophylaxis guidelines might be implemented in practice (Supplementary Methods S1.1).

We defined the fraction of all RSV cases that is preventable by a prophylaxis regimen (“preventable fraction”) to be the ratio of

- the number of RSV-associated hospitalizations that occur in a given region (state or county) while the prophylaxis regimen offers protection to those who receive prophylaxis as scheduled, and
- the total number of RSV-associated hospitalizations that occur in the same region during a single epidemiologic year.

Given that high-risk infants are a small subgroup of the general population, we assumed that our model of RSV hospitalizations among all infants is an unbiased approximation of RSV hospitalizations among high-risk infants, and therefore that the preventable fraction of a prophylactic regimen is an unbiased estimate of the fraction of high-risk infants who benefit from prophylaxis.

### Additional analyses

In addition to the 1-week rounding of our alternative prophylaxis schedules, we also calculated schedules using 2- and 4-week rounding, to assess the impact of calendrical rounding on prophylaxis performance (Supplementary Methods S1.1).

To analyze the effects of year-to-year variation between RSV seasons, we performed a similar analysis, but with the prophylaxis start date in a given year determined by the median RSV season timing during the preceding three years, rather than all years (Supplementary Methods S1.2 and S1.3).

## Results

### Timing of the RSV season varies within the state

**Figure 2:**
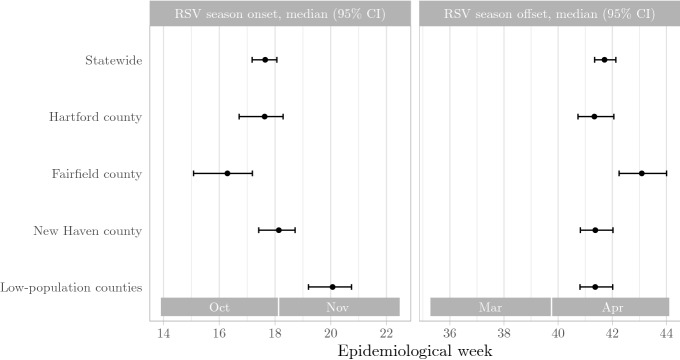
County-level variation in RSV season onset and offset in Connecticut *Figure created using *ggplot2* version 3.3.2* (https://ggplot2.tidyverse.org).

Statewide, the median onset of the RSV season is at week 17.65 (95% CI: week 17.18–18.06), and offset is at week 41.71 (95% CI: week 41.35–42.13). The earliest season onset occurred in Fairfield county, with median at week 16.29 (95% CI: week 15.08–17.19). The latest season offset also occurrent in Fairfield county, with median at week 43.09 (95% CI: week 42.25–44.01). Meanwhile, the latest season onset occurred in the low-population counties, with median at week 20.07 (95% CI: week 17.18–18.06); the season offset in these counties was not different from the statewide median (Fig. 2).

### The AAP guidelines perform best in low-population counties and worst in Fairfield county

**Figure 3:**
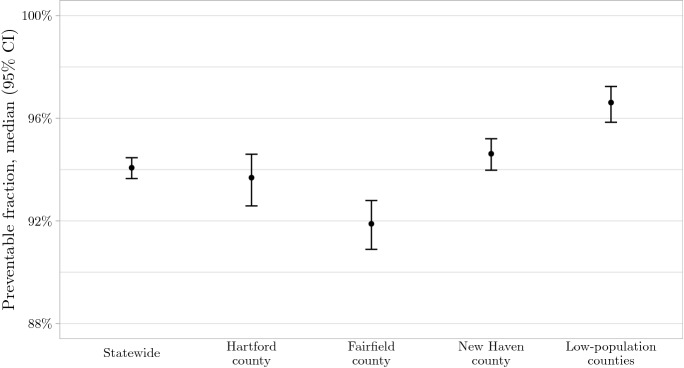
County-level variation in the preventable fraction of RSV hospitalizations according to AAP guidelines. The percentage of RSV hospitalizations occurring while protection by palivizumab—administered per the AAP guidelines—is active is plotted for each geographic region in Connecticut. *Figure created using* ggplot2* version 3.3.2* (https://ggplot2.tidyverse.org).

Statewide, our model estimates that 94.1% of RSV cases (95% CI: 93.7–94.5%) occur while palivizumab offers protection when administered per the AAP guidelines. The AAP guidelines perform better in the low-population counties, where 96.6% cases (95% CI: 95.8–97.2%) occur during the prophylaxis interval, but worse in Fairfield county, where 91.9% cases (95% CI: 90.9–92.8%) occur during this time (Fig. 3).

**Figure 4:**
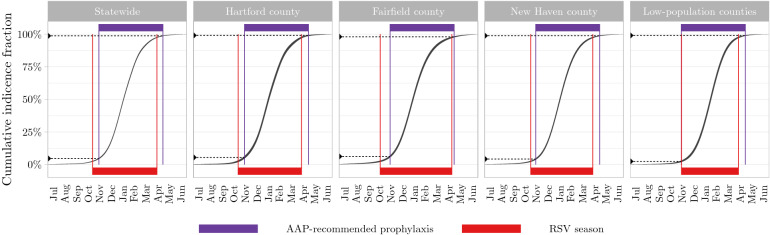
County-level variation in the relative timing of the RSV season in Connecticut and the AAP-recommended prophylaxis regimen. Comparison of the RSV season (red bar) and the prophylaxis window (purple bar) shows the misalignment between the two, notable particularly in Fairfield county. *Figure created using* ggplot2 *version 3.3.2* (https://ggplot2.tidyverse.org).

The county-to-county to variation in the protected fraction of the AAP-recommended prophylaxis is caused by temporal misalignment between the RSV season (delineated by median 2.5% onset and 97.5% offset in each county) and the timing of the AAP-recommended prophylaxis regimen (Fig. 4). Fairfield county, owing to its early season onset, sees disproportionately more cases before prophylaxis administration begins; on the other hand, the low-population counties, owing to their late season onset and relatively shorter RSV seasons, see almost all of their RSV cases within the prophylaxis window.

### Spatially adjusted regimens are superior to the AAP recommendations

**Figure 5:**
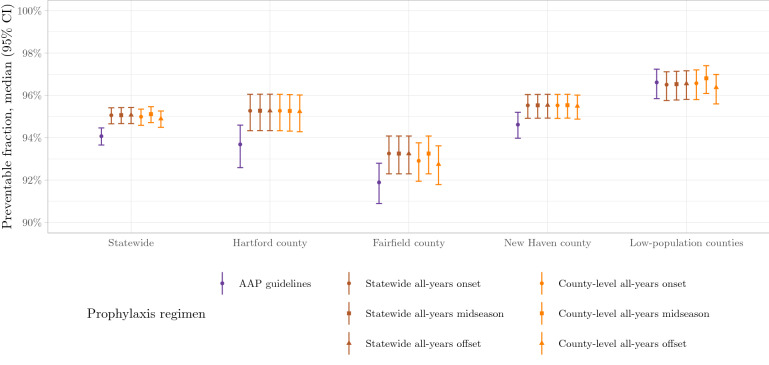
Comparison of six spatially adjusted RSV prophylaxis regimens to the AAP-recommended regimen. Spatially adjusted propxylaxis regimens are either non-inferior (for county-level analysis) or superior (for statewide analysis) to the AAP-recommended regimen. Preventable fraction: the fraction of all RSV-associated hospitalizations occurring while the prophylaxis regimen provides protection *Figure created using* ggplot2 *version 3.3.2* (https://ggplot2.tidyverse.org).

The six spatially-adjusted prophylaxis regimens yield results in all counties that are as good or better than the AAP guidelines (Fig. 5). Statewide, the AAP guidelines offer protection to 94.1% of cases (95% CI 93.7–94.5%). The most favorable of our alternative regimens—based on statewide midseason—yields an increase in the preventable fraction to 95.1% (95% CI 94.7–95.4%); other alternative regimens yield similar results. Most of the increase occurs in Hartford county, where the AAP guidelines protect 93.7% of cases (95% CI 92.6–94.6%), whereas the most favorable of our alternative regimens—also based on statewide midseason—generates an increase to 95.2% (95% CI 94.3–96.0%), with other alternative regimens being comparable. In every county, all alternative regimens are non-inferior to the AAP guidelines and non-superior to each other.

### Additional results

We also applied 2-week and 4-week rounding to the start and end dates of our six alternative immunoprophylaxis regimens, and found that doing so diminished or erased the potential benefits (Supplementary Results S1.1). Finally, we analyzed long-term trends in the RSV season onset and offset and found that the season onset may be moving earlier (Supplementary Results S1.2), but that accounting for that drift did not yield a significant further increase in preventable RSV cases (Supplementary Results S2.3).

## Discussion

Our analysis confirms the existence of county-level variation of RSV season timing in Connecticut (defined by RSV season onset and offset) observable at the county level. We have also shown a misalignment between the prophylaxis schedule recommended by the AAP and the timing of the RSV season throughout Connecticut, with most counties’ RSV season starting before protection from the first dose of prophylaxis is administered, and ending before protection from the last dose wanes. This misalignment is particularly notable in Fairfield County due to its early season onset.

Our effort to optimize timing of the prophylaxis regimen (without changing its duration and therefore its pharmaceutical cost) shows a potential $\sim 1\%$ statewide decrease in RSV-associated hospitalizations among high-risk infants each year, primarily in counties with higher RSV burden (Fairfield, Hartford, and New Haven counties). The prophylaxis regimen with which we were able to attain the best improvement is the one in which prophylaxis in each county is timed relative to the midpoint of the RSV season in that county, which simultaneously reduces unprotected cases early in the season and ineffective prophylaxis late in the season. Nevertheless, all of the alternative regimens yielded similar results, with the timing of prophylaxis adjusted in each county or statewide by less than a whole month from the current AAP guidelines. Increasing complexity of practice guidelines would carry with it increased cost of implementation (such as training of clinicians and other healthcare workers); it is therefore not apparent from our research alone that the potential benefits would outweigh the costs.

Further improvement in disease burden could likely be attained by extending the duration of the prophylaxis regimen to cover both the start and the end of the RSV season in the most populous counties (especially Fairfield county), but doing so would likely require extending the prophylaxis regimen by one additional dose and therefore increae the overall cost.

Although we found a weak statewide trend in RSV season onset becoming earlier over time, further analysis did not yield any improvements to the preventable fraction of the prophylaxis regimen based on that trend. Since temporal variation in clinical guidelines would lead to a higher implementation burden than spatial variation, we see no reason to base RSV immunoprophylaxis clinical practice guidelines on temporal variation in RSV season timing.

The use of data aggregated by epidemiological weeks created a challenge for both our analysis and the interpretation of our results. In practice, clinical guidelines are typically expressed in terms of calendar weeks or months; as a result, our analysis contains errors of up to $\pm 0.5$ weeks when applied to the more natural calendar boundaries of clinical guidelines. Ideally, our analysis should be repeated with daily case counts aggregated into weeks and months that match week and month boundaries used in clinical guidelines.

Throughout, we assumed that RSV-associated hospitalizations are an unbiased estimate of RSV incidence in the general population. In doing so, we implicitly assumed that infectiousness and virulence of RSV are constant over time, for which we have no verification.

Our model of palivizumab immunoprophylaxis assumes a constant protection period with a hard start at administration and a hard stop at 24 weeks post-administration. The physiologic response to monoclonal antibody immunoprophylaxis is actually tapered on both ends. Similarly, we assumed the protection conferred by palivizumab is constant at 100% throughout the protection period; however, not only is breakthrough RSV illness known to occur in patients on palivizumab, but it is an indication for discontinuing the prophylaxis regimen.

In areas of Connecticut associated with a relatively high amount of interstate travel (such as urban centers near the state lines), our analysis—by only considering residents of Connecticut—likely underestimates RSV burden. Finally, towns that see substantial inter-county travel (for example, employment hubs near county lines) likely bias our county-level analysis.

The method we developed for this analysis can be applied to county-level or state-level analysis of seasonal RSV patterns in other regions, some of which are likely to show a greater relative benefit (due to a greater degree of misalignment between the RSV season and the AAP guidelines’ prophylaxis window) as well as a greater absolute benefit (due to population size) than Connecticut. In particular, states of the US Upper Midwest and Pacific Northwest would likely benefit more than Connecticut, due both to their later RSV season and to the greater county-to-county differences in RSV season timing in those states^15^. A similar approach is applicable to other interventions against RSV that are sensitive to timing, including maternal vaccines and novel chemoprophylaxis, in regions where local surveillance data can be obtained.

In summary, our analysis reveals the potential for a $\sim 1\%$ gain in preventable RSV cases in Connecticut when a locally-tailored 2-week (rather than monthly) prophylaxis guideline is used in place of the established AAP guidelines. At an average of 571 RSV-associated hospitalizations per year in children under 2 years old in Connecticut, this corresponds to $\sim 5.71$ hospitalizations prevented per year, and an unknown number of prevented cases not necessitating hospitalization. Practical aspects of this potential gain—such as implementation cost—require further investigation.

## Supplementary Information

Supplementary Information 1.

Supplementary Figure 1.

Supplementary Figure 2.

Supplementary Figure 3.
