# Supplementary material for: Assessment and optimization of respiratory syncytial virus prophylaxis in Connecticut, 1996–2013
Source: Sci Rep. 2021 May 21;11:10684. doi: 10.1038/s41598-021-90107-8 (PMC8139984; doi:10.1038/s41598-021-90107-8)
Supplement: Supplementary file 1 — Supplementary Information 1. [file 41598_2021_90107_MOESM1_ESM.pdf]

Assessment and optimization of respiratory syncytial virus prophylaxis in Connecticut, 1996-2013

Ben Artin, MEng, MPH, MMSc[1,*], Virginia E. Pitzer, ScD[1], and Daniel M. Weinberger, PhD[1]

[1]Department of Epidemiology of Microbial Diseases, Yale School of Public Health, Yale University
[*]Correspondence to ben@artins.org

Supplements

S1 Supplementary methods

S1.1 Accommodating implementation complexity

We performed three variants of our spatial analysis:

- **With weekly rounding**: prophylaxis start and end date rounded to the nearest calendar week,
- **With biweekly rounding**: prophylaxis start and end date rounded to the nearest two-week calendar period, with periods chosen to approximately align with November 15th, and
- **With monthly rounding**: prophylaxis start and end date rounded to the nearest four-week calendar periods, with periods chosen to approximately align with November 15th.

Alignment of rounding periods to November 15th was done to make our regimens more clearly comparable to the AAP-recommended regimen, whose (average) start date is also November 15th. This alignment of rounding periods was only approximate because we were working with data aggregated by epidemiological weeks, which aren't aligned to calendar months.

S1.2 Temporal aggregation of RSV incidence

To analyze the effects of year-to-year variation between RSV seasons on the preventable fraction of prophylaxis regimens, we aggregated our data into (overlapping) periods of three consecutive years. We chose to use three-year periods in order to maintain a higher level of statistical power, as well as to limit the impact of short-term variation on our results. This gave us 16 three-year periods, with the 1998 period covering surveillance years 1996 through 1998, and the 2013 period covering 2011 through 2013.

We then estimated the onset and offset for each three-year period, using the same penalized spline generalized additive model as for the all-years analysis, producing — as before — statewide and county-level estimates of 2.5% onset and 97.5% offset. To evaluate year-to-year variation in RSV season onset and offset, we modeled median RSV three-year onset and offset against time using simple linear regression. We did not analyze any other long-term patterns, such as multi-year cycles.

S1.3 Evaluating alternative recent-years prophylaxis regimens

Finally, to assess the impact of any long-term trends in RSV season timing on prophylaxis, we evaluated three additional prophylaxis regimens. Here, each prophylaxis regimen is adjusted annually based on RSV data from the preceding three-year period:

- **By statewide recent-years onset**: prophylaxis administration in a given year begins at statewide median onset from the preceding three years.
- **By statewide recent-years midpoint**: prophylaxis administration begins 12 weeks before the average of the (statewide median) onset and offset from the preceding three years.
- **By statewide recent-years offset**: prophylaxis administration in a given year begins 24 weeks before the (statewide median) offset from the preceding three years.

We then compared those regimens to the one recommended by the AAP and to the six spatially adjusted alternatives (with no calendrical rounding) from our main analysis. As in the main analysis, these alternative regimens involved five monthly doses of palivizumab providing 24 weeks of protection.

S2 Supplementary results

S2.1 County-level recommendations are most useful when rounded to the nearest 1- or 2-week period

After we applied weekly, biweekly, and monthly rounding to start and end dates of our six alternative immunoprophylaxis regimens, we found that longer rounding intervals diminished the benefit of tailoring the guidelines to local data (Supplementary

figure 1). With weekly rounding, our regimens were non-inferior to the AAP recommendation, and produced an increase in preventable fraction from 94.1% (95% CI: 93.7 – 94.5%) under the AAP guidelines to 95.1% (95% CI: 94.7 – 95.4%) statewide. Biweekly rounding yielded regimens that were similarly non-inferior to the AAP recommendation, with an increase in the preventable fraction to 95.1% (95% CI: 94.7 – 95.4%) statewide. However, with monthly rounding, the gains almost completely vanished, with the preventable fraction not exceeding 94.5% (95% CI: 94.1 – 94.9%). Furthermore, all our regimens with monthly rounding were non-superior to the AAP-recommended regimen in all counties.

S2.2 RSV season onset is slowly moving earlier

Our linear regression of median RSV season onset and offset over three-year periods indicates that season onset has slowly been drifting earlier (Supplementary figure 2), at a rate of -0.91 days/year (95% CI: -2.03 – 0.21 days/year). It is unclear whether this is a part of a larger pattern, such as a cycle spanning decades. Season offset, on the other hand, has not been drifting earlier or later (95% CI: -0.81 – 0.56 days/year).

S2.3 Temporally-adjusted prophylaxis regimens are non-superior to others

Compared to our county-level all-years analysis, the three regimens based on statewide recent-years analysis produced non-superior results everywhere and inferior results in the low-population counties (Supplementary figure 3). Due to the non-superiority of regimens based on statewide recent-years analysis, we did not evaluate regimens based on county-level recent-years data.

S3 List of supplementary figures

Supplementary figure 1: The effect of calendrical rounding on prophylaxis regimens. The fraction of RSV hospitalizations occurring while a regimen offers protection is shown for each regimen and rounding interval. Longer rounding intervals diminish the advantage over the AAP-recommended regimen. Hollow plot markers (○, □, and △) denote regimens which, due to rounding, are identical to others.

Supplementary figure 2: Annual variability in RSV season onset and offset in Connecticut. While season offset has been stable, season onset has shown a statistically significant drift toward an earliear RSV season.

Supplementary figure 3: Comparison of prophylaxis regimens based on all-years data to those based on recent-years data. Recent-years analysis analysis adds no benefit over all-years analysis, for either statewide or county-level analysis. Preventable fraction: fraction of RSV hospitalizations occurring while the prophylaxis regimen offers protection
