# Supplementary figures and images for: Assessment and optimization of respiratory syncytial virus prophylaxis in Connecticut, 1996–2013
Source: Sci Rep. 2021 May 21;11:10684. doi: 10.1038/s41598-021-90107-8 (PMC8139984; doi:10.1038/s41598-021-90107-8)

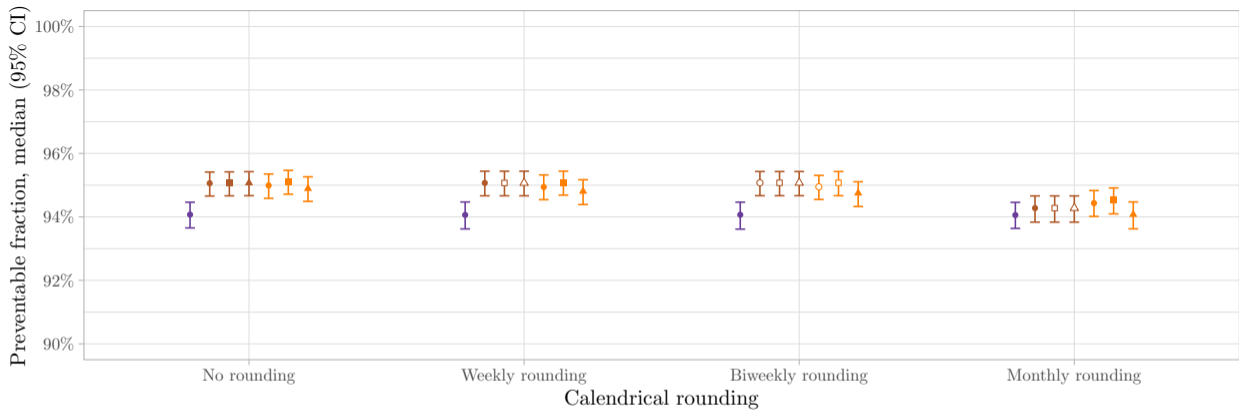

Supplement: Supplementary file 2 — Supplementary Figure 1. [file 41598_2021_90107_MOESM2_ESM.pdf]

RSV season onset, median (95% CI)

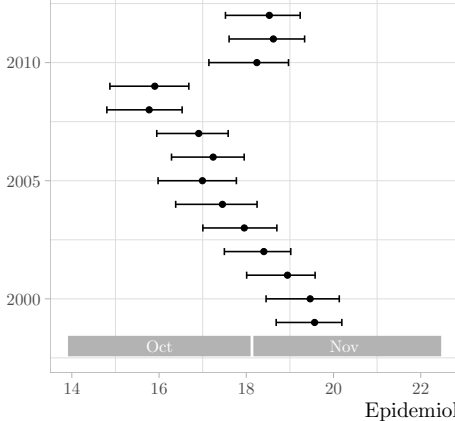

RSV season offset, median (95% CI)

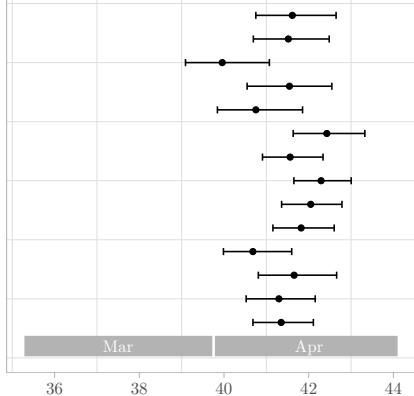

Supplement: Supplementary file 3 — Supplementary Figure 2. [file 41598_2021_90107_MOESM3_ESM.pdf]

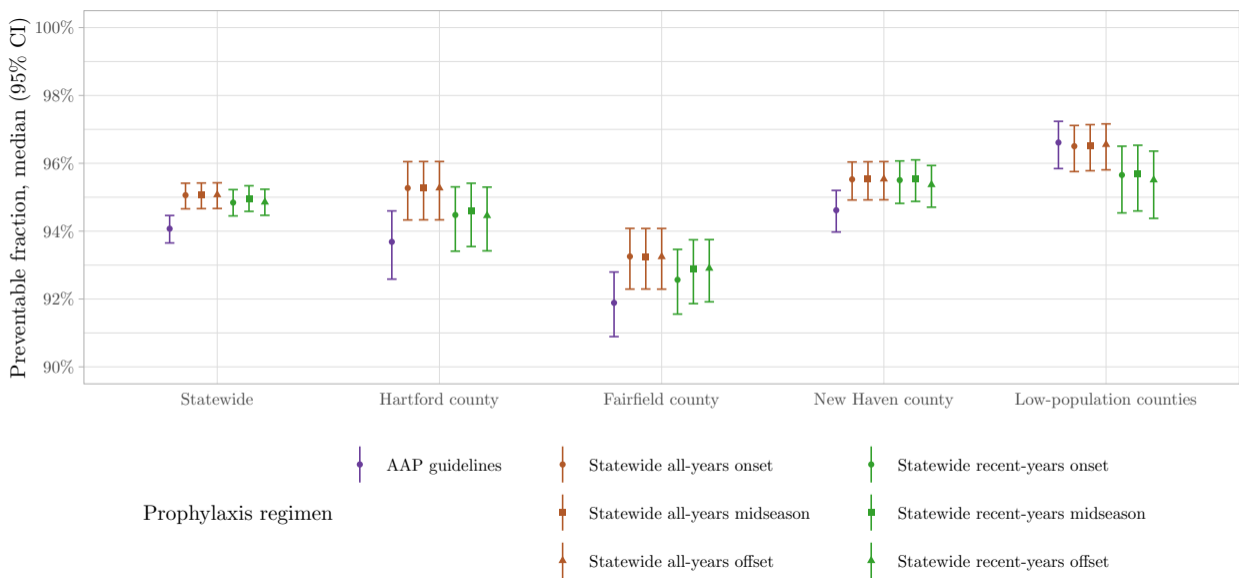

Supplement: Supplementary file 4 — Supplementary Figure 3. [file 41598_2021_90107_MOESM4_ESM.pdf]
